# Emergence and expansion of SARS-CoV-2 B.1.526 after identification in New York

**DOI:** 10.1038/s41586-021-03908-2

**Published:** 2021-08-24

**Authors:** Medini K. Annavajhala, Hiroshi Mohri, Pengfei Wang, Manoj Nair, Jason E. Zucker, Zizhang Sheng, Angela Gomez-Simmonds, Anne L. Kelley, Maya Tagliavia, Yaoxing Huang, Trevor Bedford, David D. Ho, Anne-Catrin Uhlemann

**Affiliations:** 1 Division of Infectious Diseases, Department of Medicine, Columbia University Vagelos College of Physicians and Surgeons, New York, NY USA; 2 Aaron Diamond AIDS Research Center, Columbia University Vagelos College of Physicians and Surgeons, New York, NY USA; 3 Vaccine and Infectious Disease Division, Fred Hutchinson Cancer Research Center, Seattle, WA USA; 4 Department of Microbiology and Immunology, Columbia University Irving Medical Center, New York, NY USA

**Keywords:** Microbiology, Virology, SARS-CoV-2

## Abstract

SARS-CoV-2 infections have surged across the globe in recent months, concomitant with considerable viral evolution^1–3^. Extensive mutations in the spike protein may threaten the efficacy of vaccines and therapeutic monoclonal antibodies^4^. Two signature spike mutations of concern are E484K, which has a crucial role in the loss of neutralizing activity of antibodies, and N501Y, a driver of rapid worldwide transmission of the B.1.1.7 lineage. Here we report the emergence of the variant lineage B.1.526 (also known as the Iota variant^5^), which contains E484K, and its rise to dominance in New York City in early 2021. This variant is partially or completely resistant to two therapeutic monoclonal antibodies that are in clinical use and is less susceptible to neutralization by plasma from individuals who had recovered from SARS-CoV-2 infection or serum from vaccinated individuals, posing a modest antigenic challenge. The presence of the B.1.526 lineage has now been reported in all 50 states in the United States and in many other countries. B.1.526 rapidly replaced earlier lineages in New York, with an estimated transmission advantage of 35%. These transmission dynamics, together with the relative antibody resistance of its E484K sub-lineage, are likely to have contributed to the sharp rise and rapid spread of B.1.526. Although SARS-CoV-2 B.1.526 initially outpaced B.1.1.7 in the region, its growth subsequently slowed concurrently with the rise of B.1.1.7 and ensuing variants.

## Abstract (web summary)

The dynamics of the spread of the SARS-CoV-2 variant B.1.526 suggest that resistance to neutralization by antibodies may evolve in other variants and contribute to the spread of COVID-19.

## Main

Evolution of SARS-CoV-2 was slow at the beginning of the global pandemic^6^; however, multiple major variants of concern have emerged over the past year^1–3,7^. These lineages are characterized by mutations in the spike protein, raising concerns that they may escape from therapeutic monoclonal and vaccine-induced antibodies. The hallmark mutation of B.1.1.7—a SARS-CoV-2 variant of concern first identified in the UK—is N501Y, located in the receptor-binding domain (RBD) of spike^1^. This mutation appears to render the virus more transmissible and virulent^8–10^, perhaps owing to a higher binding affinity of N501Y for the ACE2 receptor^11^ or a greater propensity to evade host innate immune responses^12^. Two other variants of concern, B.1.351^2^ and P.1^3^, also harbour the N501Y mutation, in addition to an E484K substitution in the RBD^2,3^. P.1 was identified as part of a second surge in Manaus, Brazil, despite a high pre-existing SARS-CoV-2 seroprevalence in the population^13,14^. Reinfections with P.1 and another related Brazilian variant P.2 harbouring E484K have been documented^15,16^. A previous study on B.1.351 demonstrated that this variant is refractory to neutralization by a number of monoclonal antibodies directed to the top of the RBD, including several that have received emergency use authorization^4^. B.1.351 was markedly more resistant to neutralization by plasma from individuals who had recovered from SARS-CoV-2 infection and sera from vaccinated individuals. Of note, these effects were mediated in part by the E484K mutation. These finding are concerning in light of recent reports that three vaccine trials in South Africa showed a substantial drop in efficacy^17,18^. Similarly, P.1 was also relatively resistant to antibody neutralization, although to a lesser degree^19^. In this study, we have implemented rapid molecular screening for signature mutations implicated in the success of these early variants of concern.

## Rapid screening for SARS-CoV-2 mutations

**Fig. 1:**
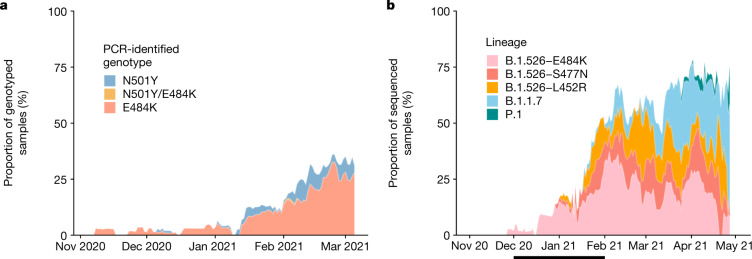
Prevalence of E484K-harbouring SARS-CoV-2 and B.1.526. **a**, Detection of viruses with key signature mutations in spike protein over time. The earliest detected variant with the E484K mutation was collected in mid-November 2020. The prevalence of E484K (samples with E484K/total PCR-genotyped samples) subsequently increased over time, from 1.8% between 1 and 15 December 2020 to 26.1% between 1 and 15 March 2021. Throughout late 2020 and early 2021, we identified fewer isolates with N501Y than with E484K, with a maximum of 5.9% of isolates containing N501Y in mid-February 2021. **b**, Distribution of different viral lineages identified by whole-genome sequencing. Within our collection of SARS-CoV-2 genomes (*n* = 1,507), the B.1.526 lineage rapidly increased in prevalence in early 2021, replacing the majority of other lineages (the blank space) present during this timeframe. This was followed by a steady rise in B.1.1.7 by mid-2021. The line below the *x* axis denotes the time period used to calculate the growth advantage of B.1.526 over other viruses that appeared earlier.

**Extended Data Fig. 1:**
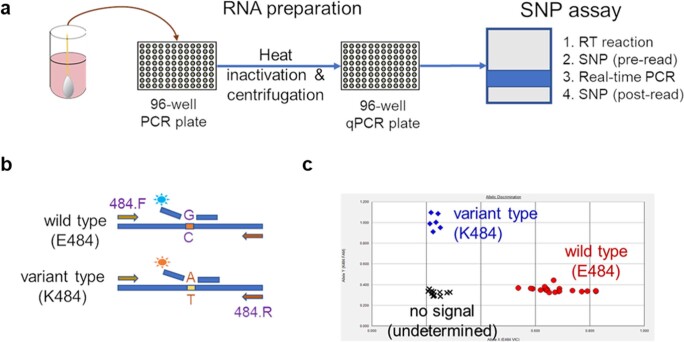
Rapid PCR-based screening assay protocol to identify samples harboring key substitutions. **a**, Viral RNA is prepared by heat inactivation and centrifugation. The supernatant is then used for the SNP assay, which entails four steps: the reverse transcription (RT) reaction, pre-PCR reading of the plate to assess background fluorescence (SNP pre-read), real-time PCR, and post-PCR reading of the plate to measure fluorescence (SNP post-read). The runtime for this entire protocol is approximately two hours. **b**, Genotype at targeted sites in COVID-19 viral RNA can be determined with two MGB probes, one for wild type (conjugated with VIC) and the other for variant type (conjugated with FAM). **c**, Example signals for the variant type (K484; blue), the wild type (E484; red) and samples with no signal (black) are shown.

We developed rapid PCR-based single-nucleotide-polymorphism (SNP) assays (Extended Data Fig. 1) to identify N501Y and E484K mutations in SARS-CoV-2-positive clinical samples stored at the Columbia University Biobank. We genotyped 1,533 samples between 1 November 2020 and 15 March 2021; 169 (11%) contained E484K, 43 (2.8%) contained N501Y and 1 sample contained both mutations. The earliest sample containing E484K was collected in mid-November 2020. The proportion of samples containing E484K increased substantially from 1.8% between 1 and 15 December 2020 to 26.1% between 1 and 15 March 2021 (Fig. 1a). Targeted PCR genotyping was continued through 1 May 2021 but was supplemented and subsequently replaced by whole-genome sequencing beginning in mid-March 2021. The frequency of viruses harbouring N501Y also increased over time, from the earliest detection in mid-January to 5.3% of screened isolates by the beginning of March.

## Genomic surveillance of SARS-CoV-2

**Extended Data Fig. 2:**
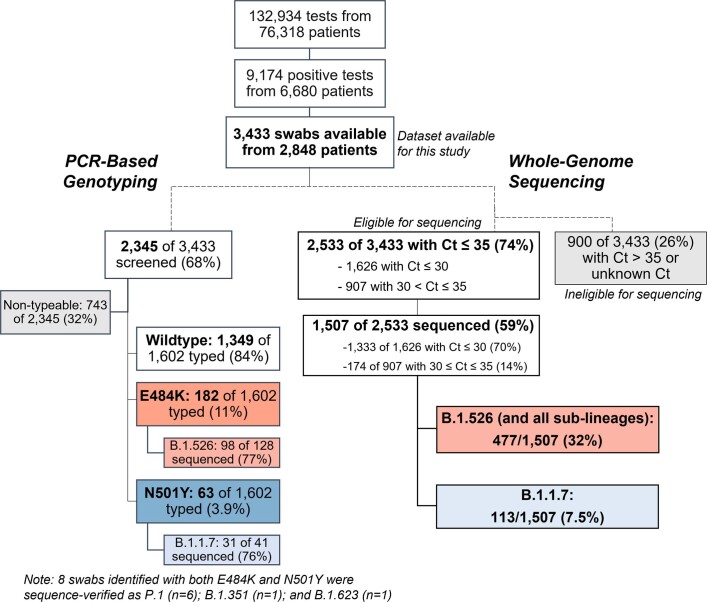
Flowchart for SARS-CoV-2-positive nasopharyngeal swabs included in this study. Top, during the study period of 1 November 2020 to 1 May 2021, 6,680 patients tested positive for SARS-CoV-2 at our hospital centre and affiliated hospitals. From these 9,174 positive nasopharyngeal swabs, 3,433 swabs were stored as part of the Columbia University Biobank COVID-19 sample repository and available for this study. Left, PCR-based genotyping assays for E484K and N501Y (see Extended Data Fig. 1) were performed on 2,345 samples. We identified a significant proportion of samples with E484K (11%), later confirmed through sequencing to primarily fall within the B.1.526 lineage, and a number of samples with N501Y (3.9%), primarily within the B.1.1.7 lineage. Right, we performed whole-genome sequencing on 1,507 samples. Of these, 32% belonged to B.1.526 and the sublineages B.1.526.1 and B.1.526.2, while B.1.1.7 constituted a much smaller proportion of samples at our centre (7.5%).

We next performed untargeted whole-genome nanopore sequencing of nasopharyngeal swab samples collected throughout the study period with a cycle threshold (*C*_t_) ≤ 35. We obtained 1,507 SARS-CoV-2 whole genomes (59% of samples with *C*_t_ ≤ 35; Extended Data Fig. 2). Sequencing results verified the E484K and N501Y substitutions in all samples identified by PCR screening. Of the sequenced N501Y isolates, 31 out of 41 (76%) were consistent with the B.1.1.7 lineage. Samples that harboured both N501Y and E484K were genotyped as P.1 (*n* = 6), B.1.351 (*n* = 1) and B.1.623 (*n* = 1). However, unexpectedly, the large majority of PCR-screened cases with E484K (98 out of 128 (77%)) were from the B.1.526 lineage^20^.

Analysis of this genomic collection (Fig. 1b) showed that by May 2021, SARS-CoV-2 variants (including B.1.526, B.1.1.7 and, more recently, P.1) comprised two-thirds of all sequenced isolates, replacing the vast majority of earlier lineages (Fig. 1b). The proportion of infections caused by B.1.526 rose rapidly from late 2020 to February 2021, and remained at approximately 40–50% of all sequenced cases from March to May 2021, despite a concurrent increase in B.1.1.7. Indeed, during December and January, when the prevalence of B.1.1.7 was still negligible (Fig. 1b), the frequency of all viruses in the B.1.526 lineage increased from less than 5% to 50%, while the frequency of other lineages declined from more than 95% to 50% (Fig. 1b, where white blank space represents other lineages). Calculations using these numbers in a head-to-head comparison and an established mathematical method^21^ indicate that B.1.526 has a growth advantage of approximately 5% per day. Similarly, fitting a logistic regression model to 478 individual observations from the extended timeframe of November 2020 to January 2021 shows that B.1.526 had a similar growth advantage of 4.6% per day (95% confidence interval 2.8–6.5% per day). Given that the serial interval for SARS-CoV-2 transmission is about 7 days in the absence of any intervention^22^, these results suggest that B.1.526 is about 35% more transmissible than non-variant viruses.

Demographic and clinical features, including clinical outcomes, were largely similar in patients infected with viruses containing E484K versus those without the signature E484K or N501Y mutations, and between patients with B.1.526-E484K versus those with non-variant lineages^23^ (Extended Data Table 1). However, significantly lower *C*_t_ values were associated with both E484K (29.49 versus 30.71, *P* = 0.013) and B.1.526-E484K (27.65 versus 28.81 in non-variant lineages, *P* = 0.015), indicating a modestly higher viral load in these variant samples. A significantly higher proportion of patients infected with B.1.526-E484K were admitted to hospital or presented to the emergency department (*P* = 0.037).

## Signature B.1.526 lineage mutations

**Extended Data Fig. 3:**
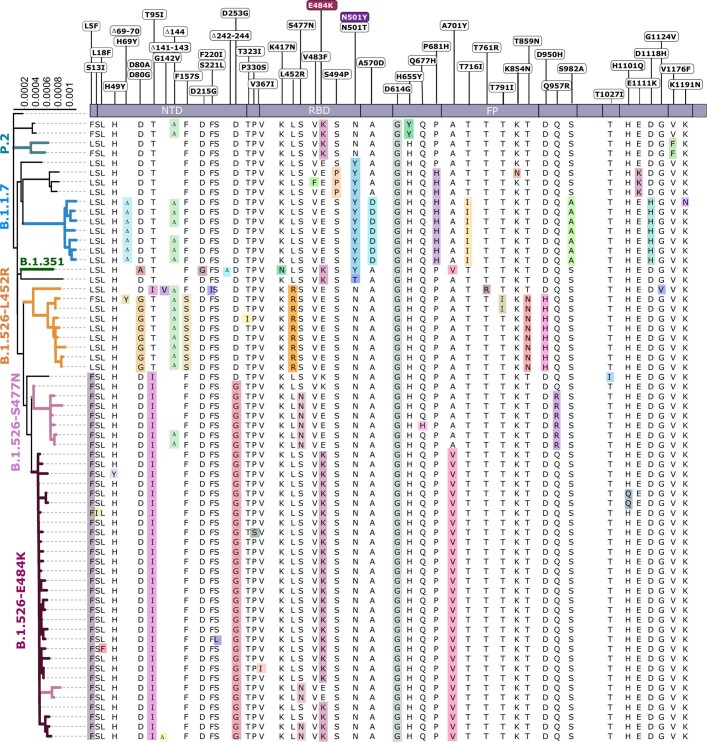
Unique patterns of spike gene mutations. Phylogenetic tree based on whole-genome alignment of genomes sequenced from our hospital centre with at least one mutation of interest or concern (E484K, N501Y, S477N, or L452R) and unique spike protein mutation constellations (*n* = 64). Branches are labelled according to Pangolin-assigned lineage identifications. Residues at which at least one sample harboured a mutation are displayed above the S-protein schematic. Residues highlighted in colour represent mutations when compared to the Wuhan-Hu-1 strain.

We identified signature spike protein mutations in the B.1.526 lineage by comparing all genomes generated in this study (Extended Data Fig. 3). Phylogenetic examination showed that the B.1.526 lineage comprises two closely related sub-lineages harbouring either E484K (B.1.526-E484K; defined as Pangolin lineage B.1.526) or S477N (B.1.526-S477N; Pangolin lineage B.1.526.2), and the additional sub-lineage B.1.526.1, harbouring the L452R substitution (B.1.526-L452R). B.1.526-E484K and B.1.526-S477N share the characteristic spike protein mutations L5F, T95I, D253G, D614G and either A701V or Q957R, along with either E484K or S477N. Non-spike mutations widely shared by B.1.526-E484K and B.1.526-S477N isolates include: T85I in ORF1a-nsp2; L438P in ORF1a-nsp4, a 9-base pair (bp) deletion (Δ106–108) in ORF1a-nsp6; P323L in ORF1b-nsp12; Q88H in ORF1b-nsp13; Q57H in ORF3a; and P199L and M234I in the N gene. While B.1.526-L452R isolates shared a number of mutations across the genome in ORF-1ab, ORF-3ab, ORF-8 and N, they did not share characteristic spike mutations with B.1.526-E484K and B.1.526-S477N.

**Fig. 2:**
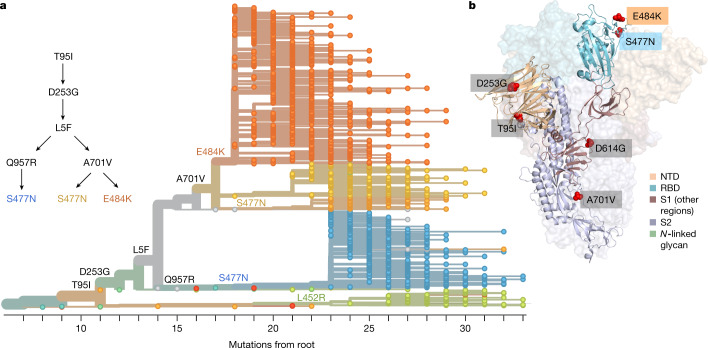
Spike protein amino acid substitutions and structural changes represented in sequenced isolates. **a**, Maximum-likelihood phylogenetic tree of 2,309 SARS-CoV-2 viruses coloured according to spike protein haplotype. Spike protein mutations are labelled on the tree, showing the stepwise accumulation of signature B.1.526 mutations T95I, D253G and L5F, and branching of B.1.526-E484K (orange) and two B.1.526-S477N sub-lineages (yellow, blue). The B.1.526-L452R sub-lineage (green) emerged in parallel. An interactive version of this figure is available at https://nextstrain.org/groups/blab/ncov/ny/B.1.526. **b**, Key mutations of B.1.526 displayed on the spike trimer. The D253G mutation resides in the antigenic supersite within the N-terminal domain (NTD), a target for neutralizing antibodies, E484K and S477N at the RBD interface with the cellular receptor ACE2, and A701V near the furin cleavage site.

To further investigate the evolutionary history of B.1.526, we performed phylogenetic analyses on genomes in this collection and in the GISAID collection harbouring the ORF1a-nsp6 deletion Δ106–108, along with the mutation A20262G that uniquely defines the parent clade containing B.1.526 and related viruses (Fig. 2a). We observed a stepwise emergence of the key lineage-defining mutations, with T95I, D253G and L5F appearing in the earliest phylogenetic nodes. Isolates subsequently branched into four sub-lineages, with two major groups B.1.526-E484K and B.1.526-S477N containing A701V, and a smaller sub-lineage B.1.526-S477N containing Q957R. The B.1.526-L452R lineage—which emerged in parallel with these—is related to B.1.526-E484K and B.1.526-S477N, but forms a distinct phylogenetic branch (Extended Data Fig. 3).

Fig. 2b shows the localization of signature B.1.526-E484K and B.1.526-S477N mutations within the spike protein. D253G resides in the antigenic supersite in the N-terminal domain^24^, which is a target for neutralizing antibodies^25^, whereas E484K is situated at the RBD interface with the cellular receptor ACE2. The A701V mutation near the furin cleavage site is also shared with variant B.1.351.

## Antibody neutralization of B.1.526

**Fig. 3:**
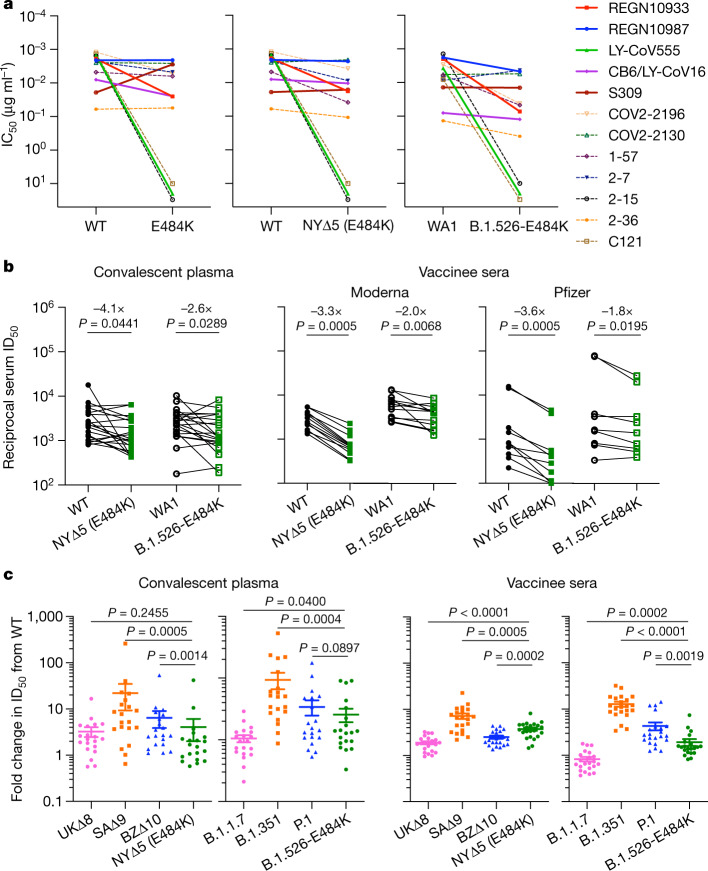
Neutralization studies of B.1.526-E484K and comparative analyses. **a**, Neutralizing activities of 12 monoclonal antibodies against pseudoviruses containing E484K alone or all five signature B.1.526 mutations (L5F, T95I, D253G, A701V and E484K) (NYΔ5(E484K)), as well as against the authentic B.1.526-E484K. Antibodies with emergency use authorization are shown with bold solid lines. Data are mean ± s.e.m. of technical triplicates and represent one of two independent experiments. IC_50_, half-maximal inhibitory concentration; WT, wild type. **b**, Fold change in virus-neutralizing activity of plasma from patients who have recovered from SARS-CoV-2 infection (convalescent plasma) (*n* = 20) and sera from vaccinated individuals (vaccinee sera) (*n* = 22) against the NYΔ5(E484K) pseudovirus compared with wild-type pseudovirus, as well as against authentic B.1.526-E484K and wild-type virus (WA1) (numbers shown above *P*-values). ID_50_, antibody dose required to reduce viral count by 50%. **c**, Fold change in neutralization ID_50_ for plasma from patients who have recovered from SARS-CoV-2 infection and sera from vaccinated individuals against different variant pseudoviruses and live viruses compared with wild-type counterparts. The data for B.1.1.7, B.1.351 and P.1 were derived from previous studies^4,19^. Data from 20 recovered patients or 22 vaccinated individuals were averaged and are presented as arithmetic mean ± s.e.m. (individual data points are also shown). Statistical comparisons were made using the Wilcoxon matched-pairs signed rank test; two-tailed *P*-values are reported.

**Extended Data Fig. 4:**
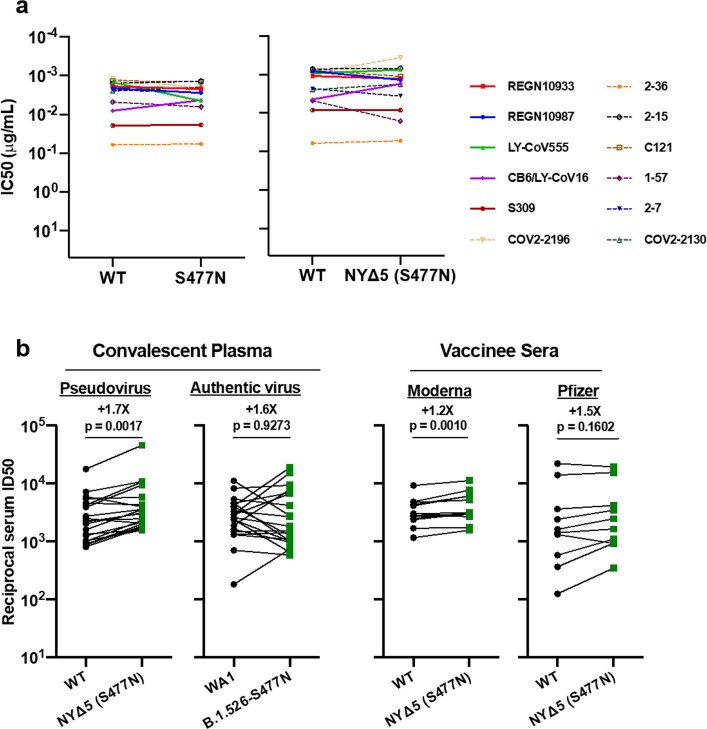
Neutralization studies of B.1.526-S477N. **a**, Neutralizing activities of 12 monoclonal antibodies against pseudoviruses containing S477N alone or all five signature B.1.526-S477N mutations (L5F, T95I, D253G, A701V, and S477N), termed NYΔ5(S477N). Antibodies with emergency use authorization are shown in bold solid lines. Data are represented as mean ± s.e.m. of technical triplicates and represent one of two independent experiments. **b**, Neutralizing activities of convalescent plasma (*n* = 20) against NYΔ5(S477N) as well as against the authentic B.1.526 virus with S477N, and neutralizing activities of vaccinee sera (*n* = 22) against the NYΔ5(S477N) pseudovirus, compared to wild-type counterparts. Statistical comparisons were made using the Wilcoxon matched-pairs signed rank test; two-tailed *P*-values are reported.

The effects of the signature spike protein mutations in B.1.526 on antibody neutralization were first assessed using vesicular stomatitis virus (VSV)-based pseudoviruses, as previously described^4,25^. Pseudoviruses were constructed containing S477N or E484K alone, or containing all five signature mutations (L5F, T95I, D253G, A701V and E484K or S477N) (NYΔ5(E484K) or NYΔ5(S477N)), and analysed in a neutralization assay with 12 monoclonal antibodies (including 5 with emergency use authorization), 20 plasma samples from patients who had recovered from SARS-CoV-2 infection and 22 sera from vaccinated individuals^4^. The neutralizing activities of 12 monoclonal antibodies covering a range of epitopes on RBD were essentially unaltered against the S477N and NYΔ5(S477N) pseudoviruses (Extended Data Fig. 4a), showing that this mutation has no discernible antigenic impact, as was validated using convalescent plasma and vaccinee sera (Extended Data Fig. 4b). However, the activities of several antibodies—including REGN10933 and LY-CoV555, which are already in clinical use—were either impaired or lost when tested against E484K and NYΔ5(E484K) pseudoviruses (Fig. 3a). Similarly, neutralizing activities of convalescent plasma or vaccinee sera were reduced by 4.1-fold or 3.3- to 3.6-fold, respectively, against NYΔ5(E484K) (Fig. 3b). Neutralization studies on the authentic B.1.526-E484K virus yielded similar results, although the magnitudes of resistance to convalescent plasma and vaccinee sera were slightly lower at 2.6-fold and 1.8- to 2.0-fold, respectively (Fig. 3b). A comparative analysis with other variants of concern (Fig. 3c) showed that antibody resistance of B.1.526-E484K is probably lower than that of B.1.351 and closer to that of P.1. Overall, these results demonstrate the need to modify the antibody therapies currently in use and to monitor the efficacy of current vaccines in regions where B.1.526-E484K is prevalent.

## The spread of B.1.526 across New York and the US

**Fig. 4:**
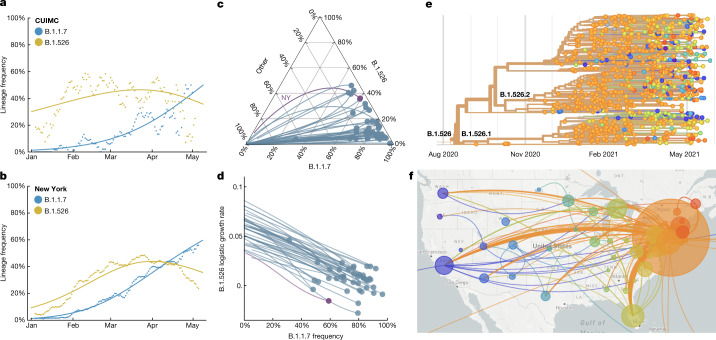
Spread of B.1.1.7 and B.1.526 lineages in New York and the United States. **a**, **b**, Frequencies of B.1.1.7 (blue) and B.1.526 (yellow) lineages in the CUIMC catchment area (**a**) and New York state (**b**) during January to May 2021, with dots representing daily seven-day sliding window averages and lines representing fit to a multinomial logistic regression model. **c**, Ternary plot of state-level frequency trajectories for 42 US states, separating frequencies of B.1.1.7, B.1.526 and other lineages. Each state-level trajectory is shown as a line from lower left in January 2021—when both B.1.1.7 and B.1.526 were rare—to right, as B.1.1.7 and B.1.526 increase in frequency. The trajectory for New York state is highlighted in purple. **d**, The same data presented in **c**, except frequency of B.1.1.7 is plotted against logistic growth rate of B.1.526. **e**, Phylogenetic tree of 933 B.1.526 samples from across the United States. Branch tips are coloured on the basis of location of sampling and branches are coloured by inferred ancestral location. **f**, Phylogeographic view of data from **e**. Each sampling location is represented as a circle with area proportional to sample count and each inferred transition event across the phylogeny is drawn as an arc connecting the inferred origin and destination. Most migration events are inferred to be direct dispersals from New York state. Data in **e**, **f** were visualized using NextStrain (https://nextstrain.org) and made available through a CC-BY-4.0 license.

Prevalence of the novel variant B.1.526 surged rapidly in the CUIMC catchment area (Fig. 4a) and throughout New York state (Fig. 4b) following its emergence in late 2020, replacing other lineages and initially outpacing B.1.1.7. A multinomial logistic regression model describing the concurrent growth rates of these two lineages shows that starting in mid-April 2021, B.1.1.7 surpassed B.1.526 owing to a slightly higher fitness, with estimated growth rates in New York state of 5.3% per day for B.1.1.7 (95% confidence interval 5.0–5.7%) and 3.4% per day for B.1.526 (95% confidence interval 3.2–3.6%) (Fig. 4b). These estimates suggest a fitness advantage of B.1.526 over existing non-variant lineages^21,22^ of 22–25% over a serial interval of 7 days during a period when multiple variants were competing simultaneously. Furthermore, the estimates also suggest a fitness advantage of B.1.1.7 over existing non-variant lineages of 35–40%, as well as a fitness advantage of B.1.1.7 over B.1.526 of 12–15%. Both lineages grew quickly (Fig. 4a, b), but once they reached a high frequency of circulating viruses, the competition between them caused the growth of B.1.1.7 to slow and that of B.1.526 to decline.

**Extended Data Fig. 5:**
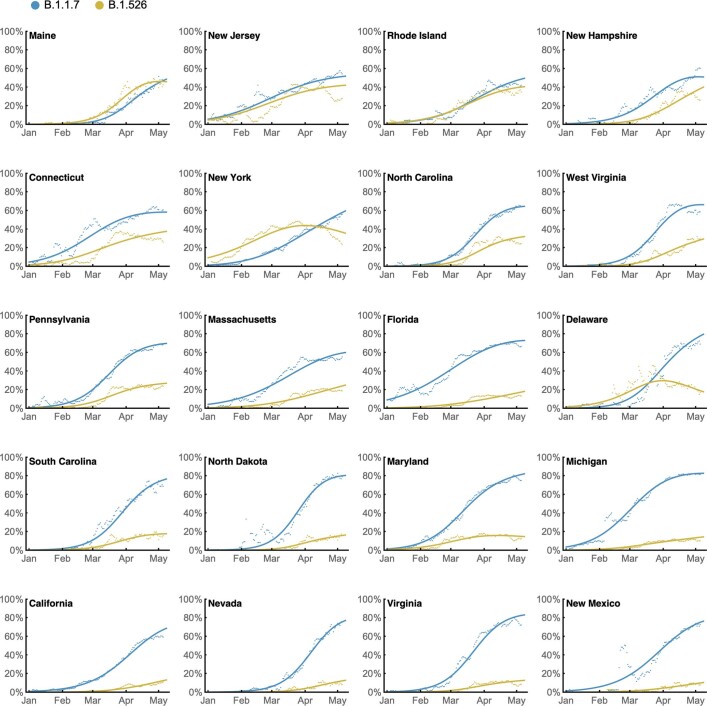
State-level growth dynamics of B.1.526 and B.1.1.7. Daily state-level frequencies of B.1.526 (in yellow) and B.1.1.7 (in blue), based on GISAID data downloaded on 6 June 2021, were used to plot 7-day sliding window averages of the prevalence of each lineage (shown as dots in the figure). A 4-parameter multinomial logistic regression model was fit directly to the observation data, in which both B.1.1.7 and B.1.526 have parameters specified for frequency at day 0 (1 January 2021) and logistic growth rate (shown as lines in the figure). States are ordered according to frequency of B.1.526 at the final timepoint of 8 May 2021.

Trajectories of the frequencies of B.1.1.7 and B.1.526 across states (Fig. 4c, Extended Data Fig. 5) show two general patterns: (1) an initial rapid increase of both lineages until the proportion of other lineages had been eclipsed, followed by a decline of B.1.526, as seen in New York and in several neighbouring states; and (2) rapid growth and resulting dominance of B.1.1.7, preventing the further rise of B.1.526. The dynamics between these two lineages is described further in Fig. 4d, which plots the logistic growth rate of B.1.526 against the frequency of B.1.1.7, again at the state level. At lower frequencies of B.1.1.7, all states show a similarly rapid growth of B.1.526 as it replaces non-variant lineages. However, as B.1.1.7 increases in frequency, the growth of B.1.526 slows, again indicative of the slightly higher fitness of B.1.1.7. At a minimum, the proportion of B.1.526 increased rapidly where B.1.1.7 was not already dominant and continued to grow at a similar pace as B.1.1.7 in several states (Extended Data Fig. 5).

Phylogeographic analysis of the B.1.526 lineage revealed ancestral viruses originating in New York in August 2020, diversifying within the state, and then dispersing to other states (Fig. 4e, f). State-level genomic data showed that B.1.526 was concentrated primarily in New York and surrounding states, including New Jersey and Rhode Island (Extended Data Fig. 5). This suggests that B.1.526—and B.1.526-E484K in particular—became widespread in the region, the original epicenter of COVID-19 in the United States^26,27^, although the lineage has also grown in states outside the northeastern United States (for example, North Carolina). By the end of April 2021, B.1.526 was widely distributed within the United States, and the lineage had emerged and expanded in multiple states across the country (Fig. 4f). This ability of B.1.526 to spread rapidly across the United States (Extended Data Fig. 5) and internationally is notable.

## Discussion

Here we report the emergence of the SARS-CoV-2 lineage B.1.526 and the surge of B.1.526 infections in New York during the second wave of the COVID-19 pandemic. Neutralization studies on B.1.526-E484K demonstrate that the activities of several antibodies are either impaired or lost with this variant, including two antibodies (Ly-CoV555 and REGN10933) that are already in clinical use. Furthermore, neutralizing activities of plasma from individuals who have recovered from SARS-CoV-2 infection or sera from vaccinated individuals were lower against B.1.526-E484K. By contrast, the S477N mutation, a key signature of another B.1.526 sub-lineage, did not have an impact on antibody neutralization.

This study has several limitations. This was a single-centre genomic survey of patients attending hospital and may not have fully captured patients with milder disease. However, our results are comparable to genomic data released by public health laboratories in the region and incorporate all publicly available data for phylogeographic context and growth rate calculations. As in all genomic surveillance studies, we predominantly sequenced samples with a *C*_t_ less than 30, but this included a high proportion of samples throughout the study period. In addition, our PCR screen enabled us to obtain unbiased estimates of E484K and N501Y prevalence early on in the study. PCR approaches may be increasingly warranted for continued surveillance during non-surge periods, during which *C*_t_ values trend higher. Finally, transmissibility estimates based on observed prevalence are imperfect as they reflect observed growth rates rather than intrinsic transmissibility of the virus.

Together, our findings underscore the importance of the E484K mutation, which has emerged in at least 246 different lineages^28^ of SARS-CoV-2, a powerful illustration of convergent evolution. This highlights that E484K can rapidly emerge in multiple clonal backgrounds and may warrant targeted screening for this key mutation in addition to robust genomic surveillance programs. However, B.1.526 is one of the few lineages with E484K that has risen to prominence. The greatest threat of B.1.526 appears to be the ease with which it spreads, with an estimated transmissibility approximately 35% higher than non-variant viruses when competing head to head. Despite the notable transmissibility of B.1.1.7, B.1.526 was able to spread rapidly in the United States, replacing other lineages and continuing to increase in frequency in several states where both B.1.526 and B.1.1.7 were predominant. Similarly, although B.1.351 may pose the greatest antigenic challenge to antibodies and vaccines, the B.1.526-E484K sub-lineage also exhibits resistance to antibody neutralization. Our findings present a clear-cut example of SARS-CoV-2 evolution in real time. B.1.526, with its higher transmissibility, appeared suddenly and rose to dominance, only to wane as variants (B.1.1.7 and, more recently, B.1.617.2) with even higher fitness emerged. These observations are a stark reminder that increasingly concerning variants are expected to emerge if SARS-CoV-2 is allowed to continue its spread.

## Methods

### Clinical cohort

This observational study took place at an academic quaternary care centre in New York City. Nasopharyngeal swabs obtained as part of routine clinical care were tested by the Clinical Microbiology laboratory, and positive specimens were transferred to the Columbia University Biobank for inactivation and storage. Electronic health records data extracted for this analysis included demographics, laboratory results, admission, discharge and transfer dates, current and historical international classification of disease codes (ICD 9 and ICD 10) extracted from the clinical data warehouse. The study was reviewed and approved by the Columbia University Institutional Review Board (IRB) (AAAT0123). The IRB waived consent for the entirety of this observational study, including for the collection and sequencing of the viral samples as well as the abstraction of the clinical metadata, as this observational study met the requirements for this exception. These include minimal risks to subjects, not adversely affecting the rights and welfare of the subjects, and that the research could not be carried out without the waiver.

### PCR screening

Extended Data Fig. 1 describes the overall protocol for variant screening. To enable rapid PCR-based screening, we prepared RNA using the heat inactivation method in place of RNA-isolation methods^29^. First, 50 µl of nasal swab sample in viral transport medium was transferred to 96-well PCR plates, covered with an adhesive aluminium foil (VWR 60941-076) and incubated at 95 °C for 5 min using the PCR instrument. After the centrifugation of the plate at >2,100*g* for 5 min, 5 µl of the supernatant from each sample, which contains viral RNA, was used for the SNP assay.

The SNP assay consists of four steps as follows: reverse transcription of viral RNA, pre-read of the SNP assay, real-time PCR and post-read of the SNP assay. 5 µl of RNA from the supernatant was added to 15 µl of the single step quantitative PCR with reverse transcription (RT–qPCR) reaction mix, which consists of 5 µl of TaqPath 1-step RT–qPCR Master Mix, CG (4×) (ThermoFisher Scientific), 500 nM of forward and reverse primers, 120 nM of VIC-MGB probe, 50 nM of FAM-MGB probe, 1/2000 volume of ROX Reference Dye (Invitrogen) as the final concentration, and nuclease-free water to adjust the total reaction volume of 20 µl. Each reaction plate included 8 control wells, 5 × 10^6^ and 5 × 10^3^ copies of WA-1 (wild type), B.1.1.7 and B.1.351, which were generated by PCR to match the variant sequences, and 2 wells with water as no template controls (NTC).

The primer pairs and probes used are as follows. For the SNP assay for position 501, a primer pair of 501.F: 5′- GGT TTT AAT TGT TAC TTT CCT TTA CA-3′ and 501.R: 5′-AGT TCA AAA GAA AGT ACT ACT ACT CTG TAT G-3′ were used with two TaqMan probes (ThermoFisher Scientific), one for wild type, VIC.N501MGB: [VIC]-AA CCC ACT AAT GGT-MGBNFQ and the other for variant type, FAM.Y501MGB: [FAM]-AAC CCA CTT ATG GT-MGBNFQ. For position 484, a primer pair of 484.F: 5′-AGA GAG ATA TTT CAA CTG AAA TCT ATCAGG-3′ and 484.R: 5′-GAA ACC ATA TGA TTG TAA AGG AAA GTA AC-3′ were used with two probes, one for wild type, VIC.E484MGB: [VIC]-ATG GTG TTG AAG GT-MGBNFQ and the other for variant type, FAM.K484MGB: [FAM]-ATG GTG TTA AAG GT-MGBNFQ.

The reaction plate was subjected to: (1) reverse transcription reaction at 25 °C for 2 min, 50 °C for 15 min and a hold at 4 °C; (2) SNP assay (pre-read) at 60 °C for 30 s; (3) real-time PCR at 95 °C for 20 s followed by 50 cycles of two-step PCR, at 95 °C for 3 s and at 60 °C for 30 s with the fast 7500 mode; followed by (4) SNP assay (post-read) at 60 °C for 30 s using ABI 7500 Fast Dx real-time PCR instrument with SDS Software (ThermoFisher Scientific). The genotype at each key position for each sample was determined by reading the component signal of the amplification and the allelic discrimination analysis software in the program.

### Whole-genome sequencing

Extended Data Fig. 2 displays a flowchart outlining samples available for this study. Isolates with *C*_t_ values below 35 were selected for sequencing using the ARTIC v3 low-cost protocol targeting 400-bp amplicons^30^ or Rapid Barcoding kit protocol targeting 1,200-bp amplicons^31^. In brief, RNA was extracted using the Qiagen RNeasy Mini kit or Zymo DNA/RNA Mini kit. Reverse transcription was performed using LunaScript RT SuperMix (NEB). Tiling PCR was performed on the cDNA, and amplicons were barcoded using the Oxford Nanopore Native Barcoding Expansion 96 kit. Pooled barcoded libraries were then sequenced on an Oxford Nanopore MinION sequencer using R9.4.1 flow cells. Base calling was performed in MinKNOW software v21.02.1. Sequencing runs were monitored in real time using RAMPART (https://artic-network.github.io/rampart/) to ensure sufficient genomic coverage with minimal runtime. Consensus sequence generation was performed using the ARTIC bioinformatics pipeline (https://github.com/artic-network/artic-ncov2019). Genomes were manually curated by visually inspecting sequencing alignment files for verification of key residues in Geneious v10.2.6.

### Phylogenetic analysis

Phylogenetic reconstruction of amino acid changes (Fig. 2a) was conducted using the Nextstrain^32^ workflow at https://github.com/nextstrain/ncov, which aligns sequences against the Wuhan Hu-1 reference using nextalign (https://github.com/nextstrain/nextclade), constructs a maximum-likelihood phylogenetic tree via IQ-TREE^33^, estimates molecular clock branch lengths via TreeTime^34^ and reconstructs nucleotide and amino acid changes (also via TreeTime). This workflow was applied to 2,309 SARS-CoV-2 genomes with the 9-bp deletion Δ106–108 in ORF1a-nsp6 along with mutation A20262G, which demarcates the parent clade to lineage B.1.526 alongside 688 global reference viruses. This analysis was conducted on data downloaded^35^ from GISAID (https://gisaid.org/) on 5 April 2021. Phylogeographic reconstruction of spread from New York state (Fig. 4e, f) was similarly conducted using the same Nextstrain workflow with the addition of performing ancestral trait reconstruction of the geographic ‘division’ attribute of 933 SARS-CoV-2 genomes downloaded from GISAID on 6 Jun 2021.

### Neutralization studies of pseudoviruses

We assayed the neutralizing activity of monoclonal antibodies, convalescent plasma and vaccinee sera against E484K, S477N and wild-type (D614G) pseudoviruses, as well as pseudovirus NYΔ5 containing all five signature mutations of B.1.526-E484K (L5F, T95I, D253G, E484K, D614G and A701V), as previously described^25^. We examined four monoclonal antibodies with emergency use authorization (CB6, REGN10987, REGN10933 and LY-CoV555), and eight additional RBD monoclonal antibodies, including from our own collection (2-15, 2-7, 1-57 and 2-36)^25^ as well as S309^36^, COV2-2196 and COV2-2130^37^, and C121^38^. We also examined convalescent plasma collected in March and April 2020 (*n* = 20 patients), and sera from individuals who had received Moderna or Pfizer vaccine^4^ (*n* = 22). In brief, Vero E6 cells (ATCC) were seeded in 96-well plates (2 × 10^4^ cells per well). Cell lines were negative for *Mycoplasma*, as assessed using the *Mycoplasma* PCR ELISA (Sigma). Pseudoviruses were incubated with serial dilutions of the test samples in triplicate for 30 min at 37 °C. The mixture was added to cultured cells and incubated for an additional 24 h. Luminescence was measured using a Britelite plus Reporter Gene Assay System (PerkinElmer), and IC_50_ was defined as the dilution at which the relative light units were reduced by 50% compared with the virus control wells (virus + cells) after subtraction of the background in the control groups with cells only. The IC_50_ values were calculated using nonlinear regression in GraphPad Prism 8.0. Statistical analysis was performed using a Wilcoxon matched-pairs signed rank test. Two-tailed *P*-values are reported.

### Neutralization of infectious SARS-CoV-2

Infectious SARS-CoV-2 isolate hCoV-19/USA/NY-NP-DOH1/2021 was isolated at the Aaron Diamond AIDS Center (Columbia University Medical Center) from a nasopharyngeal swab and propagated for one passage in Vero E6 cells (ATCC). Infectious titre of the resulting virus was determined by an end-point dilution and cytopathic effect (CPE) assay on Vero-E6 cells as described previously^25^. The virus has since been deposited at BEI Resources (catalogue (cat.) no. NR-55359). SARS-CoV-2 virus USA-WA1/2020 (WA1), obtained from BEI Resources (cat. no. NR-52281) served as the control in experiments.

An end-point dilution microplate neutralization assay was performed to measure the neutralization activity of twenty convalescent patient plasma samples and twelve purified monoclonal antibodies. In brief, plasma samples were subjected to successive fivefold dilutions starting from 1:100. Similarly, antibodies were serially diluted (fivefold dilutions) starting at 50 µg ml^−1^. Triplicates of each dilution were incubated with SARS-CoV-2 at an multiplicity of infection of 0.1 in Eagle’s minimum essential medium (EMEM; ATCC) with 7.5% inactivated fetal calf serum (FCS) for 1 h at 37 °C. After incubation, the virus–antibody mixture was transferred to a monolayer of Vero-E6 cells grown overnight. The cells were incubated with the mixture for about 70 h. CPE of viral infection was visually scored for each well in a blinded fashion by two independent observers. The results were then converted into percentage neutralization at a given sample dilution or antibody concentration, and the mean ± s.e.m. was plotted using a five-parameter dose-response curve in GraphPad Prism v8.4.

### Growth dynamics

Growth dynamics of B.1.1.7 and B.1.526 were obtained through by downloading ‘metadata’ from GISAID on 6 June 2021 for all 422,760 viruses sampled from the United States collected after 1 January 2021. These metadata have PANGO lineages^39^ already assigned to each genome sequence. Daily state-level frequencies (and frequencies for CUIMC) were extracted for plotting using seven-day sliding window averages of the prevalence of B.1.1.7 and B.1.526, calculated as the number of sequence-verified samples from each strain divided by the total number of positive samples with *C*_t_ values below 35, as this threshold value was used for sequencing. Separately, a multinomial logistic regression model was fit directly to the observation data consisting of individual genomes, their dates of sampling (independent variable *X* in days since 1 January 2021) and their category labels (dependent variable *Y*, “B.1.1.7”, “B.1.526” and “other”). This results in a 4-parameter model where both B.1.1.7 and B.1.526 have parameters specified for frequency at day 0 (1 January 2021) and logistic growth rate. This model was fit to the data using the Classify package of Mathematica v12.2.

### Reporting summary

Further information on research design is available in the Nature Research Reporting Summary linked to this paper.

## Online content

Any methods, additional references, Nature Research reporting summaries, source data, extended data, supplementary information, acknowledgements, peer review information; details of author contributions and competing interests; and statements of data and code availability are available at 10.1038/s41586-021-03908-2.

### Supplementary information

Reporting Summary

## Figures and Tables

**Extended Data Table 1:**
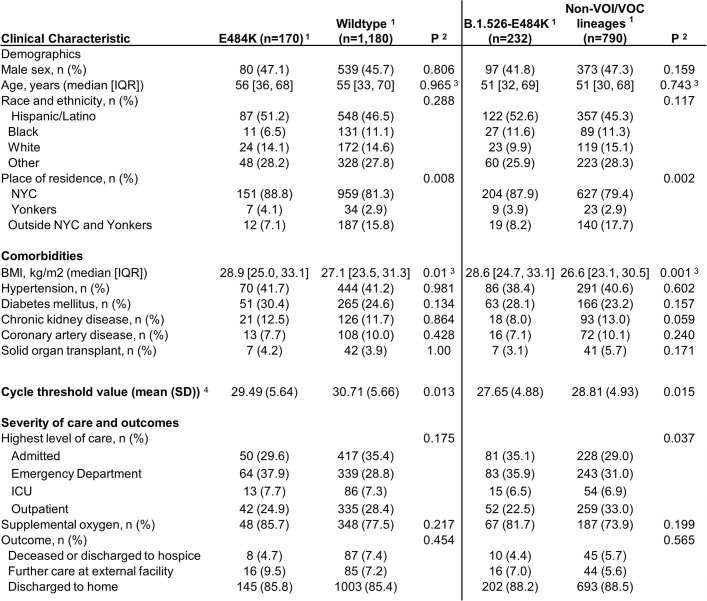
Clinical characteristics of patients infected with SARS-CoV-2 based on viral genotype

^1^Wildtype isolates are defined as those without E484K or N501Y mutations. For comparisons based on lineage, B.1.526-E484K was compared with non-variant of interest (VOI) and non-variant of concern (VOC) lineages (i.e. B.1.526.1, B.1.526.2, B.1.1.7, P.1, P.2 and B.1.351 were excluded). Comparisons were made using the first genotyped sample per patient to exclude multiple measures.

^2^T-tests (two-sided) were performed for continuous variables and chi-squared tests for categorical variables, unless otherwise indicated as below.

^3^Owing to non-normal distribution, a Kruskal-Wallis non-parametric test was used.

^4^Cycle threshold value as determined through our rapid qPCR-based screening assay on heat-inactivated nasopharyngeal swab samples.

## Data Availability

All genomes and associated metadata generated as a part of this study have been uploaded to GISAID (https://gisaid.org) and NCBI GenBank (BioProject Accession PRJNA751551). Biological materials (that is, variant pseudoviruses) generated as a part of this study will be made available but may require execution of a materials transfer agreement.

## Code availability

Data processing and visualization were performed using publicly available software and packages, primarily RStudio v1.2.5033, GraphPad Prism v8.4, and iTOL (https://itol.embl.de/). The exact workflows used for phylogenetic (Fig. 2a) and phylogeographic analysis of public GISAID data (Fig. 4e, f) are available at https://github.com/blab/ncov-ny. Frequency dynamics were modelled using Mathematica in notebooks also available at https://github.com/blab/ncov-ny.
