# Supplementary material for: Emergence and expansion of SARS-CoV-2 B.1.526 after identification in New York
Source: Nature. 2021 Aug 24;597(7878):703–8. doi: 10.1038/s41586-021-03908-2 (PMC8481122; doi:10.1038/s41586-021-03908-2)
Supplement: Supplementary file 1 — Reporting Summary [file 41586_2021_3908_MOESM1_ESM.pdf]

nature research

Corresponding author(s): Anne-Catrin Uhlemann, David D. Ho

Last updated by author(s): Jul 29, 2021

Reporting Summary

Nature Research wishes to improve the reproducibility of the work that we publish. This form provides structure for consistency and transparency in reporting. For further information on Nature Research policies, see our Editorial Policies and the Editorial Policy Checklist.

Statistics

For all statistical analyses, confirm that the following items are present in the figure legend, table legend, main text, or Methods section.

n/a	Confirmed	
☐	☒	The exact sample size (n) for each experimental group/condition, given as a discrete number and unit of measurement
☐	☒	A statement on whether measurements were taken from distinct samples or whether the same sample was measured repeatedly
☐	☒	The statistical test(s) used AND whether they are one- or two-sided<br>*Only common tests should be described solely by name; describe more complex techniques in the Methods section.*
☐	☒	A description of all covariates tested
☐	☒	A description of any assumptions or corrections, such as tests of normality and adjustment for multiple comparisons
☐	☒	A full description of the statistical parameters including central tendency (e.g. means) or other basic estimates (e.g. regression coefficient) AND variation (e.g. standard deviation) or associated estimates of uncertainty (e.g. confidence intervals)
☐	☒	For null hypothesis testing, the test statistic (e.g. F, t, r) with confidence intervals, effect sizes, degrees of freedom and P value noted<br>*Give P values as exact values whenever suitable.*
☒	☐	For Bayesian analysis, information on the choice of priors and Markov chain Monte Carlo settings
☒	☐	For hierarchical and complex designs, identification of the appropriate level for tests and full reporting of outcomes
☒	☐	Estimates of effect sizes (e.g. Cohen's d, Pearson's r), indicating how they were calculated

Our web collection on statistics for biologists contains articles on many of the points above.

Software and code

Policy information about availability of computer code

Data collection	PCR-based genotyping of nasopharyngeal swabs was performed on an ABI 7500 Fast Dx Real-Time PCR Instrument with SDS Software (ThermoFisher Scientific). Genomic sequencing was performed on an Oxford MinION and Oxford GridION and basecalling was performed using MinKNOW v21.02.1. For pseudovirus neutralization assays, SoftMax Pro v7.0.2 (Molecular Devices) was used to measure luminescence.
Data analysis	Basecalling was performed in the MinKNOW software v21.02.1. Sequencing runs were monitored in real-time using RAMPART (https://artic-network.github.io/rampart/) v1.2.0 or v1.2.1 to ensure sufficient genomic coverage with minimal runtime. Consensus sequence generation was performed using the ARTIC bioinformatics pipeline (https://github.com/artic-network/artic-ncov2019). Genomes were manually curated by visually inspecting sequencing alignment files for verification of key residues in Geneious v10.2.6. RStudio v1.2.5033, GraphPad Prism v8.4, and Mathematica v.12.2 were used for data representation and statistical analysis. Phylogenetic analyses utilized MAFFT v1.4.0 and IQ-TREE v2.0.3. The exact workflow used for phylogenetic analysis of public GISAID data (Fig. 2a, Fig. 4e-f) and frequency dynamic modeling is available at https://github.com/blab/ncov-ny.

For manuscripts utilizing custom algorithms or software that are central to the research but not yet described in published literature, software must be made available to editors and reviewers. We strongly encourage code deposition in a community repository (e.g. GitHub). See the Nature Research guidelines for submitting code & software for further information.

Data

Policy information about availability of data

All manuscripts must include a data availability statement. This statement should provide the following information, where applicable:

- Accession codes, unique identifiers, or web links for publicly available datasets
- A list of figures that have associated raw data
- A description of any restrictions on data availability

All genomes and associated metadata generated as a part of this study have been uploaded to GISAID (gisaid.org) and NCBI GenBank (BioProject Accession PRJNA751551). Biological materials (i.e. variant pseudoviruses) generated as a part of this study will be made available but may require execution of a materials transfer agreement.

Field-specific reporting

Please select the one below that is the best fit for your research. If you are not sure, read the appropriate sections before making your selection.

☒ Life sciences ☐ Behavioural & social sciences ☐ Ecological, evolutionary & environmental sciences

For a reference copy of the document with all sections, see nature.com/documents/nr-reporting-summary-flat.pdf

Life sciences study design

All studies must disclose on these points even when the disclosure is negative.

Sample size	All samples available to us at the initiation of this study were screened using our PCR-based genotyping assay (n=2,345 of 3,433 in the Columbia University Biobank repository). We selected samples with a SARS-CoV-2 cycle threshold value Ct<=35 for sequencing, due to the limitations of genomic approaches for samples with low viral load (n=1,210). For pseudovirus and B.1.526-E484K live virus assays, we obtained convalescent plasma from 20 patients, vaccinee sera from 12 Moderna SARS-Co-2 mRNA-1273 vaccine participants, and sera from 10 Pfizer BNT162b2 Covid-19 vaccine participants. This sample size is appropriate within technical capability to compare the difference between groups, as we have previously published. Phylogenetic analysis of the emergence of B.1.526 and its sublineages included 2,997 publicly available genomes from GISAID; phylogeographic analysis included 933 B.1.526 genomes from the GISAID database. Growth rate calculations per state included in total data from 422,760 genomes collected between January 1, 2021 and June 1, 2021. No statistical methods were used to predetermine sample size. All neutralization assays were performed at least in triplicate (as described below). Phylogenetic and growth dynamic analyses utilized all publicly available genomic data released at the time of this study.
Data exclusions	Genomes with greater than 8% ambiguous bases (3000 Ns) were excluded from phylogenetic analyses, based on standard criteria used in NextStrain bioinformatics pipelines and based on GISAID genome upload quality criteria.
Replication	All neutralization assays were performed with replicate measurements as indicated in the methods section and figure legends. Pseudovirus experiments in this manuscript were performed in three successful replicates prior to reporting. For the B.1.526-E484K live virus experiments, a minimum of three successful replicates were used. Neutralization assays for variants not performed in this manuscript but reported here, were performed successfully in at least triplicates and the detailed summary of those experiments have been reported in Wang et al., 2021.
Randomization	This observational study was not randomized.
Blinding	This observational study was not blinded.

Reporting for specific materials, systems and methods

We require information from authors about some types of materials, experimental systems and methods used in many studies. Here, indicate whether each material, system or method listed is relevant to your study. If you are not sure if a list item applies to your research, read the appropriate section before selecting a response.

Materials & experimental systems

n/a	Involved in the study	
☐	☒	Antibodies
☐	☒	Eukaryotic cell lines
☒	☐	Palaeontology and archaeology
☒	☐	Animals and other organisms
☐	☒	Human research participants
☒	☐	Clinical data
☒	☐	Dual use research of concern

Methods

n/a	Involved in the study	
☒	☐	ChIP-seq
☒	☐	Flow cytometry
☒	☐	MRI-based neuroimaging

Antibodies

Antibodies used	Monoclonal antibodies tested in this study were generously provided by Regeneron Pharmaceuticals (REGN10933, REGN10985, COV2-2196, and COV2-2130), Brii Biosciences (Brii-196 and Brii-198), Eli Lily (LY-CoV-555), and the Vaccine Research Center at the National Institutes of Health (CB6 - through Baoshan Zhang and Peter D. Kwong). S309 (Pinto, D. et al. Cross-neutralization of SARS-CoV-2 by a human monoclonal SARS-CoV antibody. Nature 583, 290–295 (2020)) and C121 (Robbiani, D. F. et al. Convergent antibody responses to SARS-CoV-2 in convalescent individuals. Nature 584, 437–442 (2020)) were also provided. The remaining monoclonal antibodies used in this study (1-57, 2-7, 2-15, 2-36) were produced at Columbia University as described in Liu et al. 2020 (Ref#27). Monoclonal antibodies were serially diluted (5-fold dilutions), primarily starting at 10 μg/mL, though some clinical antibodies were tested from starting concentrations of 1 μg/mL.
Validation	All of the SARS-CoV2 spike antigen-specific monoclonal antibodies obtained from commercial sources and produced in house have been thoroughly validated by epitope mapping and binding analysis to SARS-CoV-2 spike protein and neutralization experiments to SARS-CoV-2 pseudoviruses and described in our previous publications cited in this paper (Wang et al. 2021, Ref#4; Liu et al. 2020, Ref#27).

Eukaryotic cell lines

Policy information about cell lines

Cell line source(s)	Vero E6 (ATCC, Cat# CRL-1586) cell lines were used.
Authentication	Cell lines were obtained from authenticated vendors; additional authentication was not performed. Cells were recovered as healthy logarithmically growing cells within 4 to 7 days after thawing. Viability was measured and found to be >90%.
Mycoplasma contamination	All cell lines were negative for Mycoplasma, as assessed using the Mycoplasma PCR ELISA (Sigma Catalog No. 11663925910)
Commonly misidentified lines (See ICLAC register)	None

Human research participants

Policy information about studies involving human research participants

Population characteristics	Demographic and clinical characteristics for patients whose nasopharyngeal swab samples were included in this study are shown in Extended Data Table 1. Plasma samples were obtained from patients (median age 51.5; IQR 48-60; 13 males (65%) and 7 females (35%)) convalescing from documented SARS-CoV-2 infection approximately one month after recovery or later.
Recruitment	Nasopharyngeal swabs obtained as part of routine clinical care were tested by the Clinical Microbiology laboratory, and positive specimens were transferred to the Columbia University Biobank for inactivation and storage; participants were not recruited for this study. Convalescing patients volunteered for the cohort study. These cases were enrolled into an observational cohort study of convalescent patients followed at the Columbia University Irving Medical Center starting in the Spring of 2020. From their documented clinical profiles, plasma samples from ten with severe Covid-19 were selected, along with plasma from 10 with non-severe infection, for this study, as described in Wang et al. (Ref #4) and Liu et al. (Ref #27).
Ethics oversight	This study was reviewed and approved by the Columbia University Institutional Review Board (protocol number AAAT0123) and deidentified viral samples were obtained under a waiver of consent. Sera and plasma was obtained from patients after informed consent.

Note that full information on the approval of the study protocol must also be provided in the manuscript.
